# Characterization of Older Emergency Department Patients Admitted to Psychiatric Units

**DOI:** 10.14740/jocmr2311w

**Published:** 2015-09-25

**Authors:** Kirk A. Stiffler, Erol Kohli, Oriana Chen, Jennifer A. Frey

**Affiliations:** a Department of Emergency Medicine, Summa Akron City Hospital, 525 East Market Street, Akron, OH 44304, USA; b Department of Emergency Medicine, Northeast Ohio Medical University, 4209 State Rte 44, Rootstown, OH 44272, USA

**Keywords:** Emergency medicine, Geriatric, Psychiatry, Screening

## Abstract

**Background:**

Many older patients presenting to emergency departments (EDs) with psychiatric complaints require admission to geropsychiatric units (GPUs). The medical evaluation needed prior to this is not understood. Our goal was to understand ED evaluation practices for patients admitted to the GPU through the ED and understand the medical problems identified after admission.

**Methods:**

Via retrospective chart review, we abstracted demographics, medical history, ED complaint, evaluation, length of stay, and diagnosis. The number of patients later transferred from the GPU and the reasons for such transfers were also recorded.

**Results:**

Of 100 patients reviewed, the average age was 78 years. Admission diagnoses were agitation/mania (30%), depression/suicidal ideation (28%), change in mental status/confusion (12%) and other (30%). Most had at least one prior psychiatric and medical diagnosis (77%, 60%). Common ED tests ordered were basic metabolic panel (BMP) (96%), complete blood count (CBC) (94%), urinalysis (UA) (89%), electrocardiogram (EKG) (69%), alcohol level (62%), urine toxicology (61%), chest X-ray (51%), and CT scan of the head (71%). Abnormal findings included urinalysis (24.7%), CBC (23.4%), toxicology (23%), BMP (21.9%), head CT (21.1%), chest X-ray (13.7%), ECG changes (10.1%), and alcohol (4.8%). Five of the 100 GPU admissions were later transferred to a medical floor.

**Conclusion:**

Most GPU admissions have previous psychiatric and medical issues and are admitted for agitation/mania or depression/suicidal ideation. A certain percentage of patients are transferred out due to medical issues despite ED evaluation. However, it is unlikely that further ED testing would reduce this percentage. Further research of medical screening for geropsychiatric patients may elucidate ideal medical clearance procedures.

## Introduction

The number of geriatric patients seen in the emergency department (ED) for psychiatric illnesses will continue to grow as the population ages across the United States [1]. Many of these geriatric ED patients will be admitted to dedicated geropsychiatric units (GPUs). Underlying medical problems, which are the cause or a contributing factor to the illness, are occasionally identified after psychiatric admission [2]. These medical problems may include infections, dehydration, metabolic disturbances, polypharmacy, central nervous system events (ischemic or hemorrhagic), and delirium [3]. Missing delirium in the ED has been described as a medical error and an issue of quality of care [4]. Delayed and under recognition and treatment of medical conditions can lead to inappropriate admission to the GPU and ultimately adverse medical and psychiatric outcomes [3]. This is associated with higher death rates, increased health care costs, prolonged hospitalization, and accelerated long-term functional and cognitive impairment [5-7]. Currently, there is no universally accepted medical clearance evaluation for elderly patients admitted to a GPU from the ED. History and physical examinations are most commonly used to guide the emergency physician in the initial evaluation of elderly patients who are thought to require psychiatric hospitalization [8]. The goal of this study is to understand current ED evaluation practices for older patients who are admitted to the GPU and characterize the incidence of medical problems identified in the ED and after admission. This information may lead to a better understanding of what may be required to “medically clear” a geriatric patient for appropriate and safe admission to a GPU.

Our objectives were to characterize the current ED evaluation practices for patients who are admitted to the GPU, assess the incidence of medical problems identified in GPU patients who were admitted through the ED, and characterize the medical problems identified on GPU patients.

## Methods

An IRB approved retrospective chart review of 113 patients over 65 years admitted to the Summa Health System (SHS) GPU after being seen in one of three SHS EDs over a 90-day time period was performed. SHS is an adult, urban, community teaching hospital system, whose EDs see approximately 130,000 adult patients per year. The EDs included Summa Akron City Hospital, Summa St. Thomas Hospital, and the Summa Health Center at Green. Methodological strategies from Gilbert et al were applied to reliably extract data from the medical charts [9]. Investigators were trained to abstract charts for this study by using a set of sample medical records. Variables were defined precisely and data were abstracted to standard data collection forms. To blind reviewers from the relationships between the ED visit and the GPU admission, these data points were collected by different investigators. Subjects were included if they were 65 years and older and admitted from the designated ED directly to the GPU. Subjects were excluded if they were admitted to an SHS psychiatric unit within 30 days of the admission under review. The data abstracted included demographics, medical history, ED chief complaint and evaluation including laboratory testing and diagnostic imaging, ED diagnosis, and the number of patients transferred out of the GPU for medical reasons. A single data manager was designated to resolve any data discrepancies. A sample size of 100 patients provides 95% confidence intervals ± 10% for dichotomous variables. Data were analyzed using Microsoft Excel^TM^ and STATA^®^ to determine means and proportions.

## Results

**Figure 1:**
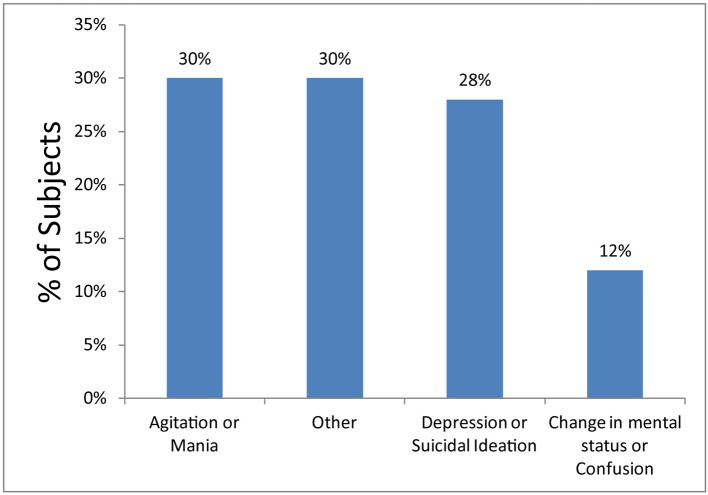
Chief complaints of geropsychiatric patients in the ED. Other chief complaints included debility, medication noncompliance, anxiety, paranoia, hallucinations, delusions, and behavioral disturbance.

In this retrospective sample of 113 patients admitted to the GPU between July 2012 and September 2012, 13 patients were excluded due to psychiatric admission in the 30 days prior, leaving a study size of 100 patients. The average patient age was 78 years (range 65 - 99 years) 58% were female, and 89% were Caucasian. ED chief complaints were grouped into agitation/mania (30%), depression/suicidal ideation (28%), change in mental status/confusion (12%), and other (30%) (Fig. 1). The majority of subjects had a least one prior psychiatric diagnosis (77%) and at least one other prior medical diagnosis (60%). Subjects took an average of 9.4 medications (range 1 - 26).

The most common ED tests ordered were basic metabolic panel (BMP) at 96%, complete blood count (CBC) at 94%, urinalysis (UA) at 89%, CT scan of the head without contrast at 71%, electrocardiogram (EKG) at 69%, alcohol level at 62%, toxicology screen at 61%, and chest X-ray at 51%. Abnormal findings identified in the ED (from most frequent to least) included UA (24.7%), CBC (23.4%), toxicology screen (23%), BMP (21.9%), head CT (21.1%), chest X-ray (13.7%), EKG changes (10.1%), and alcohol level (4.8%) (Table 1). However, of those patients with positive toxicology screens, it is important to note that a number of patients were prescribed opiates and/or benzodiazepines accounting for a fraction of the positive test results.

**Table 1:** ED Diagnostic Testing

Diagnostic test	Subjects tested (n = 100)	Abnormal tests	If ED action was taken for abnormal tests
Basic metabolic panel	96%	21.9% (21/96)	14.3% (3/21)
Complete blood count	94%	23.4% (22/94)	0% (0/22)
Urinalysis	89%	24.7% (22/89)	11.2% (10/89)
Head CT scan without contrast	71%	21.1% (15/71)	0% (0/15)
Electrocardiogram	69%	10.1% (7/69)	0% (0/7)
Alcohol level	62%	4.82% (3/62)	0% (0/3)
Toxicology screen	61%	23.0% (14/61)	0% (0/14)
Chest X-ray	51%	13.7% (7/51)	0% (0/7)

**Figure 2:**
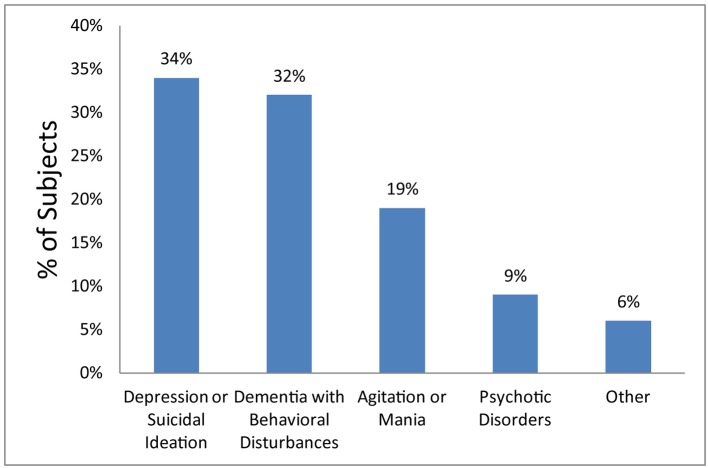
Admission diagnoses of geropsychiatric patients. Other admission diagnoses included delirium, functional decline, decompensated mental health disorder, schizophrenia, asymptomatic bacteriuria, and mild hyponatremia.

GPU admission diagnoses included depression/suicidal ideation (34%), dementia with behavioral disturbances (32%), agitation/mania (19%), psychotic disorders (9%), and other (6%) (Fig. 2). Five percent were later transferred from GPU to a medical floor: one for persistent leukocytosis and acute on chronic renal insufficiency, one for sepsis secondary to urinary source, one for hematemesis, one for dehydration and concern for neuroleptic malignant syndrome, and one for chest pain.

## Discussion

While there have been numerous studies describing the “medical clearance” of psychiatric patients in the ED, we have been unable to find any studies documenting the psychiatric medical clearance specific to the geriatric population. Given the demographics of our aging population, we suspect the number of geriatric patients presenting to the ED with acute psychiatric illness will continue to rise. With our initial study findings, we hope to offer additional insight and increase awareness about this unique subset of patients presenting to the ED.

Physicians must remember that the history and physical exam can successfully identify a majority of medical problems and substance use in ED psychiatric patients. However, in the geriatric population, the presentation can be more vague and non-specific. Furthermore, geriatric assessment is more complex than the standard medical evaluation due to increased co-morbidities, medication use and variable baseline functional status [10]. Even for patients who have a previous psychiatric history or diagnoses, appropriate medical clearance must be done to evaluate for underlying medical conditions that may be exacerbating or contributing to the psychiatric decompensation. Despite the relatively extensive medical evaluation many of the patients underwent in this review prior to admission to the GPU, several patients still required subsequent transfer out of the GPU secondary to medical issues.

ED testing yielded abnormal results across a variety of studies, including from most common to least common: UA, CBC, toxicology screen, BMP, head CT, chest X-ray, ECG and alcohol. It is important to note that for the majority of these findings, the ED physician did not take action (Table 1). We believe this is largely due to chronic diseases, including but not limited to: chronic anemia, non-specific mild electrolyte abnormalities or renal insufficiency, chronic cardiomegaly on chest X-ray, chronic ECG abnormalities such as bundle branch blocks, and atrophy with chronic findings on head CT. For acute illnesses including UTI, action was taken by the ED physician and included antibiotic treatment in addition to often sending urine cultures. BMP abnormalities acted upon were most often hypokalemia, treated with oral replacement therapy while in the ED.

It is our opinion that the medical clearance undertaken for these GPU candidates was likely adequate, as only one of the five transfers from the GPU due to medical reasons could have possibly been prevented. This patient was initially admitted to the GPU for mania and transferred to the medical floor 1 week later for worsening renal function and leukocytosis with blood cultures positive for MSSA bacteremia. After 6 days of medical floor management including antibiotics, repeat blood cultures which were negative, and a surgical consultation for a soft tissue hand wound requiring non-surgical local wound care only, this patient was transferred back to the GPU for continued behavioral abnormalities. On review of the ED chart of this patient, an elevated creatinine (Cr) of 2.88 (baseline Cr 1.49 approximately 2 years ago) was the only anomaly noted. This elevation was thought to be from dehydration by the ED physician. No fever, leukocytosis, or clinically significant hand wound was noted in the ED.

Of the remaining four transfers out of the GPU, none appear to be preventable upon evaluation of their ED clearance and clinical course. The patient who developed urinary sepsis while in the GPU was noted to have a UTI in the ED, was treated with antibiotics, and had a urine culture sent. This patient had no evidence of systemic illness by objective criteria in the ED prior to admission. The patient who was transferred out because of hematemesis did not develop this issue until day 10 of admission, and had a normal hemoglobin level during their ED evaluation. The patient transferred out for concern of neuroleptic malignant syndrome developed such symptoms on day 7 of GPU admission. Chest pain developed in one other patient during the first day in the GPU, requiring transfer to telemetry for cardiac ischemia evaluation, which was found to be negative. The ECG for this patient done in the ED as part of medical clearance evaluation was unchanged from all prior ECGs, and the patient had no cardiac related complaints noted in the history or found on examination in the ED.

In summary, we believe that of these five patients who were transferred to the medical floor, only the first patient described who developed worsening renal function and MSSA bacteremia could have been potentially identified based on the ED examination and medical clearance procedures undertaken and thus admitted to a medical floor instead of the GPU for medical management. Other transfers out of the GPU are likely related to the ongoing complex medical management issues of geriatric patients in general rather than being related to missed opportunities for diagnosis in the ED. Given this information, we believe that our current medical screening process for geropsychiatric patients (though not standardized) is likely adequately sensitive.

Several limitations in this study limit the overall generalizability. The sample size of only 100 patients seen in a single health care system makes it difficult to draw generalized conclusions. By grouping chief complaints and ED diagnoses, we may lose certain subgroups of psychiatric illness in the elderly population. The use and documentation of a standardized screening test such as the mini-mental state examination while difficult in a busy ED, may have allowed for a more accurate and standardized comparison among study subjects. While our study focused on the initial ED presentation and screening of geriatric patients admitted to the GPU, it did not focus on ED treatment and stabilization of such patients. Furthermore, if the patient had a medical illness identified during their ED evaluation as causing or contributing to their psychiatric complaint, they would have been admitted to a medical service (i.e. acute coronary syndrome, stroke, pneumonia, or acute electrolyte disturbances) and therefore not included in this retrospective review.

Nonetheless, in this study of 100 patients, the majority of ED patients admitted to the GPU were found to be white females with previously diagnosed psychiatric and medical issues, on a multitude of medications, and admitted for depression/suicidal ideation or dementia with behavioral disturbances. Patients admitted to the GPU often have an extensive evaluation in the ED prior to their admission. Despite this ED evaluation, a certain percentage of patients are still transferred out of the GPU at a later point for medical reasons. However, it is unlikely that any additional testing or evaluation beyond what is currently performed in our EDs would have a substantial impact on reducing transfers out of the GPU. Further research regarding potential standardization of medical screening with associated sensitivities, specificities, and associated costs for such screening will help identify best practices for admission to GPUs.
